# Source of Strength and Relational Catalyst Support: Pathways to Personal Growth and Thriving Among Sexually and Gender-Diverse Young Adults

**DOI:** 10.3390/bs16071096

**Published:** 2026-07-02

**Authors:** Cora R. Baron, Nancy L. Collins, Brooke C. Feeney

**Affiliations:** 1 Department of Psychological and Brain Sciences, University of California, Santa Barbara, Santa Barbara, CA 93106, USA; ncollins@ucsb.edu; 2 Department of Psychology, Carnegie Mellon University, Pittsburgh, PA 15213, USA; bfeeney@andrew.cmu.edu

**Keywords:** personal growth, thriving, LGBTQ+, close relationships, social support, well-being

## Abstract

Personal growth is a central aspect of development and well-being during young adulthood, yet sexually diverse and gender-diverse (SGD; a more inclusive term for LGBTQ+) young adults navigate this process within unique contexts shaped by identity, stress, and varying levels of social support. Despite growing visibility and social recognition of SGD identities in the United States, SGD individuals continue to face prejudice and discrimination, which negatively affects their physical and psychological health. Research indicates that stigmatized and marginalized populations with greater psychosocial resources are better able to cope with identity-related stressors. Yet, scholarship on coping with stigma and discrimination remains largely disconnected from research on social support, personal growth, and thriving within close relationships. The present observational study of SGD young adults (*N* = 400) examines how identity-affirming support from close others contributes to positive well-being outcomes, specifically personal growth, self-concept clarity, and thriving. Whereas much prior work focuses on how support buffers stress, we examine its role across stressors and opportunities for growth, experienced broadly and in relation to SGD identity. Our findings underscore the critical role that close relationships play in fostering social safety and personal growth for SGD young adults navigating identity development.

## 1. Introduction

All young people, regardless of sexual orientation or identity, deserve a safe and supportive environment in which to achieve their full potential.

—Harvey Milk

Personal growth is a key element of thriving, particularly in young adulthood. For LGBTQ+ (or sexually and gender-diverse; SGD) young adults, the search for meaningful growth experiences intersects with unique developmental experiences including identity exploration, coming out, navigating family and social acceptance, and establishing authentic self-expression within often heteronormative environments. While roughly 23% of Generation Z adults in the U.S. now identify as LGBTQ+ (Gallup, 2026), increased visibility has not eliminated the prejudice they face in many aspects of daily life (American Civil Liberties Union, 2026).^1^ These inequities point to an urgent need to understand the factors that foster personal growth and well-being among sexually and gender-diverse communities, particularly during the transition to young adulthood.

The term SGD (sexually and gender-diverse) describes people who are not exclusively heterosexual and/or whose gender identity or expression differs from their sex assigned at birth.^2^ Young adulthood is a time when SGD individuals are solidifying their sense of identity and shifting who they rely on for emotional support. Researchers have long characterized adolescence and young adulthood as a critical window for identity formation. Foundational models conceptualize this developmental window as a critical time for ego growth, where individuals transition toward coherent adult identities (Erikson, 1968). Young adulthood is also marked by a reorganization of attachment relationships, as attachment functions gradually shift from parents to peers and romantic partners, with parents remaining as “attachment figures in reserve” (Hazan & Zeifman, 1999; Hazan & Shaver, 1990). For SGD young adults, however, this developmental picture is complicated by the possibility that parental support—particularly support that affirms identity—may be absent, limited, or conditional (Diamond & Alley, 2022).

Broadly, SGD populations are recognized as a health-disparate population, facing a heightened burden of specific health conditions (e.g., depression, cardiovascular disease) and barriers to inclusive and affirming healthcare (National Institutes of Health, 2024). Recent data from the U.S. Centers for Disease Control and Prevention (CDC, 2024) demonstrates that these disparities begin in young adulthood: SGD adolescents face worse health outcomes than their cisgender and heterosexual peers, including higher rates of bullying, suicidal ideation, depression, and substance use. A growing body of theoretical work seeks to identify the individual, social, and structural mechanisms underlying these disparities.

Two theoretical perspectives guide SGD health disparity research. The minority stress model (Meyer, 1995, 2003) examines disparities through the lens of social stress and marginalization. Conversely, the social safety model identifies insufficient social safety—a lack of reliable inclusion and protection—as the primary driver of health disparities (Diamond et al., 2021; Diamond & Alley, 2022). Reflecting these frameworks, the U.S. Department of Health and Human Services’ “Healthy People 2030” initiative targets the reduction in adverse outcomes like substance use, suicidal ideation, and bullying among sexual and gender minorities (U.S. Department of Health and Human Services, 2024). While preventing these adverse outcomes is vital, investigating factors that promote health, personal growth, and thriving is equally essential.

What processes contribute to thriving among SGD young adults? Thriving involves both effectively coping with adversity and progressing toward personal growth and self-discovery. Growing evidence suggests that individuals with strong psychosocial resources may better manage identity-related stressors (McConnell et al., 2016; Poteat et al., 2020) and more actively benefit from positive identity experiences (Feinstein et al., 2020). A key psychosocial resource is social support from close others that is caring, understanding, and validating (Caprariello & Reis, 2011). Supportive relationships should help SGD individuals cope with minority stress while also fostering personal growth, identity exploration, and well-being. Broadening beyond a deficit-focused lens to include these positive developmental processes provides a more complete picture of SGD well-being.

The goal of the present study is to explore how two types of social support—source of strength and relational catalyst support (Feeney & Collins, 2015a)—collectively predict resilience, personal growth, and other components of thriving among sexually and gender-diverse young adults. In doing so, we draw from Baron and Collins’s (2025) model of thriving in SGD populations, which integrates work on minority stress, social safety, and thriving through relationships (Diamond & Alley, 2022; Feeney & Collins, 2015a; Meyer, 1995). Guided by this framework—which emphasizes the shared experiences of navigating stigma and cultivating social safety across the collective SGD community—our study focuses on this single, unified group of emerging adults rather than testing divergent pathways or differences between specific identity subgroups. This approach allows us to theoretically capture how these foundational support mechanisms operate across the broader community.

### 1.1. Social Stress and Health in Sexually and Gender-Diverse Populations

Sexually and gender-diverse (SGD) young adults experience distinct stressors beyond those of their heterosexual and cisgender peers (Major et al., 2018). Researchers have identified three relevant aspects of identity-related stress. The first is identity concealment, or hiding one’s identity to avoid stigma, which is positively correlated with mental health symptoms (Pachankis et al., 2020) and particularly challenging during young adult identity formation. The second is identity disclosure, or the high-stakes process of coming out to others; during young adulthood, fear of rejection is magnified by ongoing dependence on social and family networks (Ryan et al., 2015). Third are heteronormativity and cisnormativity—belief systems that presume heterosexuality and cisgender identity as the default (Nadal et al., 2016; Van Der Toorn et al., 2020). These assumptions create chronic stress because they are embedded within education, healthcare, and legislative systems (Burton et al., 2021; Hatzenbuehler, 2016; Toomey et al., 2013). Most existing SGD research has centered on these identity-specific stressors.

SGD young adults show elevated rates of anxiety, depression, mood disorders, substance use, and suicidality compared to their heterosexual and cisgender counterparts (Cochran et al., 2003; Hatzenbuehler, 2011; Hoy-Ellis, 2023). Emerging evidence also suggests that physical health consequences accumulate from chronic stress, including increased risk of cardiovascular disease, sleep disturbances, and systemic inflammation (Bonomo et al., 2024; J. Campbell et al., 2024; Diamond et al., 2021); and that the cascading effects of childhood and adult victimization among sexually diverse adults predict negative health outcomes in later adulthood (Alley et al., 2023). Two leading theoretical models guide scholarship on SGD health disparities: the minority stress model and the social safety model (for review, see Baron & Collins, 2025).

#### 1.1.1. Minority Stress Model

The *minority stress model* explains SGD health disparities as the product of chronic stress from identity-related stigma and discrimination (Frost & Meyer, 2023; Meyer, 1995, 2003). SGD populations encounter forms of minority stress that are common to other marginalized groups, as well as stressors distinct to their community. These include external stressors (prejudice, discrimination, and social rejection) and internal stressors (internalized stigma, expectations of rejection, and identity concealment)—both of which are especially salient during young adulthood, a period of identity consolidation and shifts in attachment figures. Stigma can produce harmful responses at both the individual (e.g., affective, cognitive, behavioral, or physiological changes) and the community (e.g., systematic exclusion in education, healthcare, and housing; Major et al., 2018) levels. Ultimately, this chronic stress is linked to somatic symptoms, maladaptive coping, reduced access to health-promoting resources, and elevated allostatic load—cumulative “wear and tear” on the body (McEwen, 1998). Building on this foundation, Diamond and Alley (2022) introduced the social safety model, which emphasizes the absence of social safety as a key mechanism impacting minority health.

#### 1.1.2. Social Safety Model

The social safety model suggests that health disparities are driven not only by minority stress, but also by the chronic lack of social safety (Diamond & Alley, 2022). Social safety can be broadly defined as a reliable sense of acceptance, belonging, inclusion, and protection. When SGD young adults lack consistent signals of social safety, they enter a state of chronic threat-vigilance that depletes their cognitive and emotional resources during a stage of critical socioemotional development. Insufficient social safety can also increase the activation of self-protective behaviors such as social withdrawal and identity concealment, which may buffer immediate social threats but ultimately undermine long-term thriving. Consequently, the social safety model argues that mitigating health disparities requires moving beyond simply reducing active discrimination to proactively cultivating environments where individuals feel fundamentally valued, secure, and structurally protected.

### 1.2. Social Support Processes in SGD Young Adulthood

#### 1.2.1. Studies of Support Processes in SGD Young Adulthood

There is a limited but growing literature on support processes in SGD young adulthood. This work often conceptualizes social support as a buffer against the harmful effects of minority stress (Meyer, 2015). Reviews demonstrate that social support is a protective factor for SGD youth (Hall, 2018; McDonald, 2018). Numerous studies demonstrate a negative association between social support and mental health symptomatology (e.g., anxiety, depression) for SGD adults and adolescents (Abreu et al., 2024; Budge et al., 2014; Doty et al., 2010; Snapp et al., 2015; Watson et al., 2019). Additionally, longitudinal work finds a robust link between lack of familial support and psychological distress in SGD youth (McConnell et al., 2016).

Despite these promising insights, several notable gaps remain in the literature. Existing work examines social support primarily in the context of minority stress, leaving its role in identity exploration, personal growth, and other positive life experiences largely unexplored. Additionally, prior research has focused heavily on reducing adverse mental health outcomes such as anxiety and depression (Hatzenbuehler & Pachankis, 2021), with less attention given to support as a tool to foster positive well-being and thriving. Despite social support being a well-established predictor of well-being among SGD young adults, the specific interpersonal mechanisms by which close relationships serve as a protective factor are not well understood.

The current study draws from contemporary perspectives on social support and close relationships to better understand how supportive relationships can benefit SGD populations in both the presence and absence of stress. According to Feeney and Collins (2015a, 2015b), close relationships support thriving through more than simply coping with hardship—they also function to foster personal growth and exploration in the absence of adversity.

#### 1.2.2. Thriving Through Relationships

Feeney and Collins (2015a) proposed a thriving through relationships model, which identifies two life contexts in which social support helps people flourish: coping with adversity and pursuing opportunities for personal growth. Drawing from attachment theory (Bowlby, 1979; Collins & Feeney, 2000), this framework views social support as an interpersonal process with two distinct support functions. The first, source of strength (SOS) support, operates during times of stress or hardship, where relationships serve as a safe haven, offering protection and stability to help people cope, recover, remain resilient, and even grow through adversity. The second, relational catalyst (RC) support, facilitates exploration and growth in the absence of adversity. This involves providing a secure base that encourages curiosity, goal pursuit, and the development of new skills and perspectives. Importantly, the model conceptualizes supportive relationships not only as a buffer against stress and hardship, but as active contributors to personal growth and long-term thriving (Deci & Ryan, 2000; Diener, 2006).

The thriving through relationships approach defines thriving across five interconnected dimensions: hedonic well-being (happiness, life satisfaction), eudaimonic well-being (personal growth, progress toward meaningful goals), psychological well-being (self-acceptance, resilience, mental health), social well-being (positive relationships and relational expectations), and physical well-being (sleep, fitness, physical health). This multidimensional framework offers a comprehensive approach for understanding how different aspects of thriving might be impacted by (a) the setbacks SGD young adults commonly face, such as managing discrimination or navigating the coming out process, and (b) the opportunities unique to their experiences, such as exploring gender expression or forming same-gender relationships—and the presence or absence of social support in each context.

The thriving through relationships model also outlines the biopsychosocial mechanisms through which SOS and RC support produce long-term thriving. Receiving support, or perceiving that it is available, can trigger immediate changes in emotions, self-perceptions, how events are appraised, motivation, behavior, relationship quality, physiological responses, and health habits (Feeney & Collins, 2015a). Accumulated over time, these event-based shifts are proposed to give rise to more enduring outcomes such as personal growth, positive self-views, physical health, resilience, and satisfying relationships. When integrated with models of minority stress and social safety, this framework provides a foundation for understanding how supportive relationships can be a vital cue for social safety in SGD young adults.

### 1.3. Current Research

The social safety model views consistency and reliability as central to the experience of social safety. It describes how individuals can perceive both safety-related signals—such as feelings of connectedness, inclusion, affirmation, and protection—and threat-related signals—such as devaluation, exclusion, and rejection—across multiple layers of their social world, spanning family, peers, schools, workplaces, and broader communities (Diamond & Alley, 2022). Baron and Collins (2025) propose that cues of social safety are embedded, explicitly and implicitly, within interpersonal support exchanges. Specifically, these cues are conveyed through SOS support, when loved ones help one another cope with stress, and through RC support, when they encourage identity exploration, personal growth, and self-discovery.

The current research is guided by Baron and Collins’s (2025) model of thriving among SGD populations, which integrates work on thriving through relationships (Feeney & Collins, 2015a) and social safety (Diamond & Alley, 2022). Specifically, we examine how the receipt of both SOS and RC support shapes appraisals of life events—both stressors and opportunities for growth—and predicts positive well-being among SGD young adults (ages 18–25). Using new data from a cross-sectional study, we examine associations between self-reported distress related to recent stressors, engagement in current goals, self-efficacy for coping with stressors, self-efficacy for pursuing goals, and multiple domains of well-being over the past month.

First, we examine the unique and shared roles of SOS and RC support in the context of both stressors and opportunities for personal growth. We distinguish between stressors and goals that affect the general population and stressors and goals specific to the SGD experience. We hypothesize that young adults receiving higher levels of SOS support will report lower distress associated with stressful life events—both general and identity-specific—and greater perceived self-efficacy for coping with these challenges. Furthermore, we hypothesize that those receiving more RC support will report greater pursuit of personal goals and growth opportunities across both general and identity-specific domains, and greater perceived self-efficacy for achieving those goals.

Second, we examine the unique roles of SOS and RC support in predicting distinct components of positive well-being, differentiating between resilience-related and growth-oriented outcomes. We conceptualize SOS support—support that provides emotional and instrumental aid during times of adversity—as particularly relevant for resilience-related outcomes, including environmental mastery, interpersonal trust, self-acceptance, and a broader sense of felt security. In contrast, we conceptualize RC support—support that affirms and facilitates goals, aspirations, and identity development—as especially important for growth-oriented outcomes, such as personal growth, purpose in life, self-concept clarity, and pride in one’s LGBTQ+ identity.

Accordingly, we hypothesize that SOS support will uniquely predict resilience-related aspects of thriving, whereas RC support will uniquely predict growth-related aspects of thriving. At the same time, these two forms of support are theoretically interrelated and are expected to jointly contribute to resilience, goal pursuit, and well-being. To capture these patterns, we adopt a theory-guided and data-driven approach to identify the specific components of well-being most strongly associated with each type of support, allowing us to examine both their unique and overlapping contributions.

## 2. Materials and Methods

The present study used a cross-sectional survey methodology with a sample of 400 sexually and/or gender-diverse young adults aged 18 to 25 years. All study procedures and materials were approved by our university’s IRB (73-23-0343).

### 2.1. Participants

Participants were recruited through Prolific, an online platform that enables individuals to participate in anonymous research studies for compensation. To be eligible, participants were required to reside in the United States, be between 18 and 25 years old, and self-identify as sexually and/or gender-diverse (LGBTQ+) at the time of the study. Of the 445 enrolled participants, 45 were excluded due to age ineligibility (e.g., indicating that they were over 25 in the comments field) or substantial missing data (>50% missing), resulting in a final analytic sample of 400 participants (*M* age = 22.6 years, *SD* = 1.9).^3^ Sample demographic characteristics are presented in Table 1.

Participants’ sexual and gender identities were assessed using a comprehensive set of demographic items assessing gender identity, transgender identity, and sexual orientation, including structured response options and opportunities to self-describe. Approximately half of participants (54.3%) self-identified as women, with the remaining identifying as men (29.3%), non-binary (14.3%), or another gender identity. The most common sexual orientation identity was bisexual+ (51.3%), and 18.8% of participants identified as transgender. The sample included participants from a range of racial and ethnic backgrounds: 50.1% Non-Latino/a White, 15% Multiracial, 13.5% Asian, 12% Black, 8.3% Latino/a, and 1.1% other or preferred to self-describe. Approximately half of the sample (46%) were students.

### 2.2. Procedure

Eligible participants received a Qualtrics survey link, which began with an embedded consent form. Consenting participants completed a survey assessing general and identity-specific stressors and goals, social support, and well-being over the past month. The survey took approximately 15 min to complete, and participants received $3 in compensation.

### 2.3. Measures

#### 2.3.1. Stressors, Source of Strength Support, and Self-Efficacy for Coping

*Stressors.* To assess participants’ responses to recent stressors, they were asked to reflect on general and identity-specific stressors experienced over the past 30 days. Participants were then presented with a list of 30 stressors developed for this study, along with an open-ended option to describe and rate an additional stressor not represented in the list.^4^ Some stressors reflected a general life domain (e.g., “feeling dissatisfied with my career,” “difficulties at work or school”), whereas others were specific to SGD identity (e.g., “feeling stigmatized as a result of my LGBTQ+ identity,” “having to lie or conceal my LGBTQ+ identity”). Participants rated the extent to which they had been affected by each stressor in the past 30 days on a 5-point Likert scale from 1 (*not at all affected*) to 5 (*extremely affected*). Items were averaged to compute an overall stress appraisal score across all 30 listed items and, when provided, the additional self-generated stressor (α = .92). An SGD identity–related stress subscale was computed from the nine SGD identity-specific stressors (α = .87).

*Source of Strength (SOS) Support.* Source of strength support was assessed by asking participants to rate how frequently close others (e.g., friends, family, romantic partners) provided various types of support during conversations about stressful experiences over the past month. Using the prompt, “In the past 30 days, when I talk about stressors, my close others have…”, participants evaluated 20 behaviors adapted from Feeney and Collins (2015a) to assess key aspects of SOS support. Responsive behaviors included items such as “made me feel understood,” “validated my thoughts, feelings, and perspectives,” and “validated and accepted all aspects of my identity.” Unresponsive behaviors (e.g., “seemed disinterested,” “criticized me or blamed me for the problem”) were reverse-scored. All items were rated on a 5-point Likert scale from 1 (*never)* to 5 (*always*) and averaged to create a composite score (α = .95).

*Self-Efficacy for Coping with Stressors.* Perceived coping self-efficacy was assessed using seven items adapted from prior work (Chesney et al., 2006; Cohen et al., 1983; Schwarzer & Jerusalem, 1995) to gauge the capability to handle current stressors, sense of control, and resilience. The instructions asked participants to evaluate their feelings regarding recent stressors over the past 30 days. Sample items included “I am capable of overcoming these stressors,” “I believe I have the skills and resources to cope with these challenging situations,” and “I feel like difficulties are piling up so high right now that I cannot overcome them” (reverse-scored). Items were rated on a 6-point Likert scale from 1 (*strongly disagree*) to 6 (*strongly agree*) and averaged to create a composite score (α = .92).

#### 2.3.2. Goals, Relational Catalyst Support, and Self-Efficacy for Achieving Goals

*Goals.* To assess participants’ responses to current goals and opportunities for growth, they were asked to reflect on their personal goals, plans, and opportunities for growth and discovery over the past 30 days. They were then presented with a list of 14 goals, plans, and life opportunities developed for this study (see Note 4), along with an open-ended option to describe an additional goal or opportunity not represented in the list. Some items reflected general life domains (e.g., “finding a good job or career,” “finding friendships,” “learning a new hobby or skill”), whereas others were specific to SGD identity (e.g., “building confidence within my LGBTQ+ identity,” “being open and authentic about my LGBTQ+ identity”). For each item, participants rated the extent to which they had focused on pursuing or thinking about it in the past 30 days on a 5-point Likert scale ranging from 1 (*not at all focused on*) to 5 (*extremely focused on*). Items were averaged to compute a general goal pursuit score across all 14 listed items and, when provided, the additional self-generated goal (α = .83). An SGD identity–related goal pursuit subscale was computed from the four SGD identity-specific goals (α = .90).

*Relational Catalyst (RC) Support.* Relational catalyst support was assessed by asking participants to rate how frequently close others (e.g., friends, family, romantic partners) provided various types of support during conversations about their goals or life opportunities over the past month. Using the prompt, “In the past 30 days, when I talk about my goals or life opportunities, my close others have…”, participants evaluated 19 behaviors adapted from prior work (Feeney & Collins, 2015a) to assess key features of RC support. Responsive behaviors included items such as “showed genuine interest and enthusiasm,” “encouraged me to pursue my goals,” and “helped me develop a plan of action.” Unresponsive behaviors (e.g., “seemed disinterested,” “demeaned or dismissed my goals”) were reverse-scored. Items were rated on a 5-point Likert scale from 1 (*never*) to 5 (*always*) and averaged to form a composite score of responsive RC support (α = .94).

*Self-Efficacy for Achieving Goals.* Perceived self-efficacy for achieving goals was assessed using seven items adapted from generalized self-efficacy frameworks (e.g., Schwarzer & Jerusalem, 1995) to capture participants’ confidence in their ability to pursue and attain currently relevant goals and life opportunities. Collectively, the items capture confidence (versus self-doubt) in one’s capacity to make progress toward personal goals, persist despite obstacles, and maintain motivation and focus. The instructions asked participants to evaluate their feelings regarding recent goals and life opportunities over the past 30 days. Sample items included “I am capable of achieving my goals,” “I am capable of making the most of these life opportunities,” and “I feel like my goals are out of reach” (reverse-scored). Items were rated on a 6-point Likert scale from 1 (*strongly disagree*) to 6 (*strongly agree*) and averaged to create a composite score (α = .89).

#### 2.3.3. Components of Thriving

*Positive Well-Being*. Positive well-being was assessed using the Psychological Well-Being (PWB) Scale (Ryff & Keyes, 1995; Ryff & Singer, 2006). This 54-item measure includes six subscales (9 items each): autonomy (e.g., “I have confidence in my opinions, even if they are contrary to the general consensus”; α = .81), environmental mastery (e.g., “In general, I feel I am in charge of the situation in which I live”; α = .88), personal growth (e.g., “I think it is important to have new experiences that challenge how you think about yourself and the world”; α = .85), positive relations with others (e.g., “People would describe me as a giving person, willing to share my time with others”; α = .86), purpose in life (e.g., “Some people wander aimlessly through life, but I am not one of them”; α = .83), and self-acceptance (e.g., “When I look at the story of my life, I am pleased with how things have turned out”; α = .91). Each item was rated on a 6-point Likert scale ranging from 1 (*strongly disagree*) to 6 (*strongly agree*).

*Self-Concept Clarity.* Self-concept clarity was assessed using the 12-item Self-Concept Clarity Scale (J. D. Campbell et al., 1996), which measures stability and clarity of one’s sense of self, values, and beliefs. Sample items included “In general, I have a clear sense of who I am” and “My beliefs about myself seem to change very frequently” (reverse-scored). We supplemented with three additional items developed for this study to better capture clarity regarding identity (e.g., “I have a clear sense of my identity”). Items were rated on a 5-point Likert scale from 1 (*strongly disagree*) to 5 (*strongly agree*) and averaged to compute a composite score (α = .93).

*LGBTQ+ Pride.* Pride was assessed using three items developed for this study measuring pride and comfort in one’s identity (e.g., “I feel proud to be LGBTQ+” and “I feel comfortable talking to people about my LGBTQ+ identity”). Items were rated on a 5-point Likert scale from 1 (*not at all true of me*) to 5 (*very true of me*) and averaged to create a composite score (α = .87).

*Connection to LGBTQ+ Community.* Connection was assessed using three items developed for this study measuring belonging and engagement (e.g., “I feel a sense of belonging and acceptance within the LGBTQ+ community” and “I feel disconnected from other LGBTQ+ people” [reverse-scored]). Items used the same 5-point scale and were averaged to create a composite score (α = .73).

## 3. Results

The primary goal of this study was to examine the unique and shared roles of SOS and RC support in predicting responses to life stressors, opportunities for growth, and distinct components of positive well-being. Descriptive statistics and the full bivariate correlation matrix for all study variables are presented in Table 2.

### 3.1. Unique Roles of SOS and RC Support in Predicting Stressors and Opportunities

**Figure 1:**
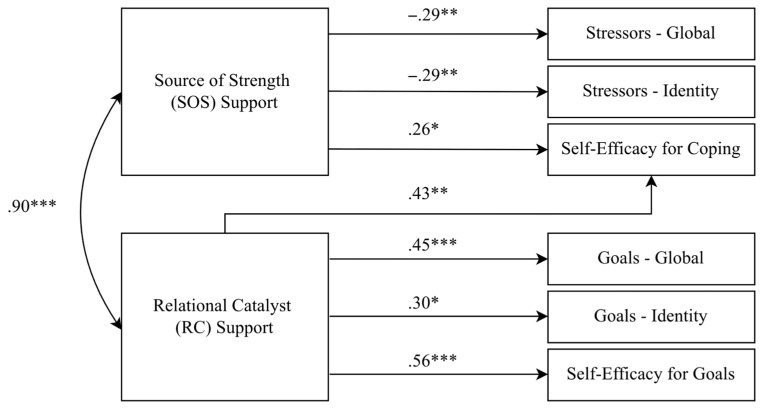
Summary of source of strength and relational catalyst support predicting responses to stressors and opportunities for growth. Path coefficients are standardized regression coefficients (*β*s). * *p* < .05. ** *p* < .01. *** *p* < .001.

To examine whether SOS and RC support uniquely predicted responses to stressors and life opportunities, we conducted a series of simultaneous multiple regression analyses. Given the high correlation between the two predictors (*r* = .89) and our goal of disentangling their unique and shared contributions, we conducted multicollinearity diagnostics and commonality analyses alongside the regression models. The Variance Inflation Factor (VIF) for all models was 4.3, falling below the conservative threshold of 5.0 and confirming that standard errors remained stable enough to reliably partition independent effects. Commonality analyses further decomposed each model’s explained variance into the portion uniquely attributable to SOS support, the portion uniquely attributable to RC support, and the portion common to both. Results are presented in Table 3 and summarized visually in Figure 1.

*Responding to Stressors.* For global stressors, higher SOS support was significantly associated with lower distress (*β* = −.29, *p* = .001), whereas RC support was not a unique predictor (*β* = .05, *p* = .567). Commonality analysis confirmed this asymmetry: although 72% of the explained variance was common to both support types, the remaining unique variance was carried almost entirely by SOS support. Similarly, for identity-related stressors, receiving higher levels of SOS support predicted significantly lower distress (*β* = −.29, *p* = .009), but RC support did not (*β* = .11, *p* = .352), with the unique variance again concentrated in SOS support. For coping self-efficacy, receiving higher levels of both SOS (*β* = .26, *p* = .043) and RC support (*β* = .43, *p* = .001) predicted greater confidence in one’s ability to cope, with commonality analysis indicating that both support types contributed meaningfully to the unique, as well as shared, variance in this outcome.

*Responding to opportunities for growth*. For global goal pursuit, higher RC support was associated with increased goal pursuit (*β* = .45, *p* < .001), whereas SOS support was not uniquely related (*β* = −.17, *p* = .066); commonality analysis showed that RC support accounted for the majority of unique variance (45%), while SOS support’s unique contribution was negligible. Similarly, for identity-related goal pursuit, higher RC support predicted increased goal pursuit (*β* = .30, *p* = .041) and accounted for 30% unique variance, while SOS support was not a unique contributor (*β* = −.062, *p* = .656). Finally, for goal attainment self-efficacy, higher RC support predicted greater confidence in one’s ability to achieve goals (*β* = .56, *p* < .001), again carrying the unique variance, whereas SOS support was not a significant predictor (*β* = .10, *p* = .367).

Together, these findings indicate that while a substantial portion of explained variance was shared between SOS and RC support across stressor and goal-related outcomes, each support type also accounted for unique variance in a distinct subset of outcomes: SOS support uniquely predicted lower distress in response to stressors, while RC support uniquely predicted greater engagement with growth opportunities.

### 3.2. Unique Roles of SOS and RC Support in Predicting Distinct Components of Thriving

A series of simultaneous regressions evaluated the unique contributions of SOS and RC support to individual components of psychological well-being, self-concept clarity, and LGBTQ+ identity variables, again accompanied by commonality analyses (Table 4). As in the prior analyses, most explained variance was common to both support types (79% to 89% across outcomes); however, each support type retained a smaller but theoretically meaningful unique component.

Controlling for SOS support, RC support was uniquely and positively related to all six components of psychological well-being such that higher levels of RC support were associated with greater personal growth (*β* = .46, *p* < .001), purpose in life (*β* = .33, *p* = .006), autonomy (*β* = .27, *p* = .019), environmental mastery (*β* = .38, *p* = .003), self-acceptance (*β* = .49, *p* < .001), and positive relations with others (*β* = .29, *p* = .005). In contrast, controlling for RC support, SOS support was uniquely related only to positive relations with others (*β* = .57, *p* < .005), with marginal positive associations for purpose in life (*β* = .21, *p* = .06) and self-acceptance (*β* = .25, *p* = .06). Commonality analyses reinforced this pattern: although the large majority of explained variance was shared between the two support types, RC support consistently accounted for the larger share of unique variance across most well-being domains, while SOS support’s unique contribution was more circumscribed, concentrated primarily in the relational outcome.

For self-concept clarity, higher SOS support was significantly associated with greater clarity (*β* = .44, *p* < .001), but RC support was not a significant unique predictor (*β* = .02, *p* = .875). Commonality analysis confirmed that, while the majority of the explained variance was shared, nearly all of the unique variance in this outcome was attributable to SOS support.

Finally, as predicted, higher levels of RC support uniquely predicted greater LGBTQ+ pride (*β* = .45, *p* = .003) and stronger connection to the LGBTQ+ community (*β* = .42, *p* = .002), whereas SOS support was not a significant unique predictor for either pride (*β* = .20, *p* = .184) or community connection (*β* = .11, *p* = .407). Here too, the majority of the variance was shared by both forms of support, but the unique variance was concentrated almost entirely in RC support.

### 3.3. Latent Variable Path Analysis

Finally, to provide a theory-guided conceptual synthesis of our regression findings, we specified a latent variable structural equation model (SEM) using the *lavaan* package in R (Version 4.6.0). Because our numerous well-being outcomes are highly intercorrelated, this approach allowed us to organize them into broader, theoretically meaningful latent constructs while simultaneously estimating the unique predictive paths of SOS and RC support on each construct. This approach both synthesizes the pattern of findings across outcomes and guards against the inflated Type I error associated with conducting many separate regression analyses.

To empirically evaluate our theoretical distinction between resilience and growth-related outcomes, we first conducted an exploratory factor analysis. Based on empirical and theoretical alignments, the measurement model specified four latent dimensions: *Growth* was defined by two indicators, personal growth and purpose, representing the extent to which individuals perceive themselves as developing positively over time and pursuing meaningful goals. *Self-integrity* was defined by autonomy and self-clarity, capturing the degree to which individuals act in accordance with their values and hold a clear, coherent understanding of themselves. *Security* was defined by positive relations with others, self-acceptance, and environmental mastery. This dimension reflects a foundational sense of security, rooted in positive models of self and others and a sense of competence engaging with the broader world. *Identity* was defined by two indicators: pride in LGBTQ+ identity and a sense of connection to the LGBTQ+ community. This reflects a novel dimension of well-being specific to SGD identity and rooted in positive feelings about one’s identity as affirmed within the larger SGD community.

Following Anderson and Gerbing’s (1988) two-step approach, we first evaluated the measurement model for our outcome variables via a baseline Confirmatory Factor Analysis (CFA) wherein all latent constructs were freely correlated, and the two observed predictors were allowed to covary with the latent factors. The initial measurement model yielded a significant absolute fit index, *χ*^2^ (31) = 212.30, *p* < .001, which is known to be overly sensitive to small discrepancies in large samples (Hu & Bentler, 1999; Kline, 2023). While the RMSEA (0.120, 90% CI: [0.106, 0.137]) was initially elevated, the incremental and residual metrics demonstrated acceptable baseline fit (CFI = .94; SRMR = 0.058).

To localize the source of this absolute misfit, we inspected the measurement model’s modification indices. We followed a sequential, theory-guided process of specifying residual covariances between individual item indicators one at a time. After each addition, indices were reassessed, retaining only those that were both statistically indicated and theoretically justifiable due to conceptual overlap between indicators. This iterative process resulted in the specification of four residual covariances within the measurement model. This final measurement model exhibited good-to-excellent fit across indices: *χ*^2^(27) = 99.663, *p* < .001; RMSEA = 0.082, 90% CI [0.065, 0.100]; CFI = .974; SRMR = 0.041. The RMSEA remained marginally above the conventional threshold of 0.08, though the lower bound of the confidence interval (0.065) is consistent with close fit.

**Figure 2:**
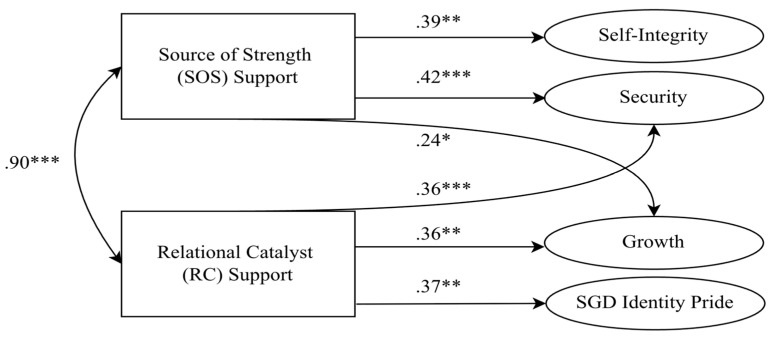
Summary of latent variable model results predicting four components of well-being from source of strength and relational catalyst support. Path coefficients are standardized regression coefficients (*β*s). * *p* < .05. ** *p* < .01. *** *p* < .001.

In the second step, we specified our primary structural model by directing paths from our two observed predictors to the four latent outcomes. Because this structural regression layer was fully saturated, the directional paths accounted for zero additional degrees of freedom, maintaining the exact global fit metrics established in the final measurement model. Crucially, the final structural path estimates remained robust and uninflated by the measurement-level modifications. Parameter estimates for the final model are presented in Table 5, and a visual summary of key regression paths is displayed in Figure 2.

Our primary aim was to examine the unique associations between the two types of support (SOS and RC) and the four latent dimensions of well-being. As hypothesized, the two relational sources of support displayed distinct, specialized roles across the outcomes. Higher perceived SOS support was associated with a stronger sense of Security (*β* = .42, *p* < .001) and greater Self-integrity (*β* = .39, *p* < .001). SOS support also emerged as a significant, but less pronounced, predictor of Growth (*β* = .24, *p* = .044), but was not uniquely related to Pride (*β* = .13, *p* = .332). In contrast, higher perceived RC support was associated with greater identity-related Pride (*β* = .37, *p* = .004), higher Growth (*β* = .36, *p* = .006), and a stronger sense of Security (*β* = .36, *p* < .001). Contrary to expectations, RC support was not a unique predictor of Self-integrity (*β* = .05, *p* = .657).

## 4. Discussion

The transition to young adulthood represents a critical developmental window characterized by identity exploration, the pursuit of life purpose, and emerging autonomy. For SGD young adults, this developmental trajectory is navigated within a unique and often hostile sociopolitical climate (Baron & Collins, 2025; Diamond & Alley, 2022). Structural stigma, minority stress, and interpersonal rejection frequently threaten the psychological well-being of SGD individuals. Historically, relationship science examining marginalized youth has operated from a deficit-based framework, focusing predominantly on risk mitigation and coping with adversity. While understanding risk is vital, it leaves an incomplete picture of SGD development.

The current study sought to address this gap by shifting the lens toward thriving in terms of positive well-being and personal growth. Integrating the thriving through relationships model of social support (Feeney & Collins, 2015a) with models of minority stress and social safety (Brooks, 1981; Diamond & Alley, 2022; Meyer, 2003), we evaluated how distinct relational support functions collectively and uniquely contribute to multi-dimensional well-being among SGD young adults. Using a latent variable SEM approach, we established four distinct, theoretically driven dimensions of thriving: identity, growth, self-integrity, and security. Our primary structural hypotheses were supported, revealing both specialized and shared contributions of SOS and RC support systems in fostering different components of thriving.

### 4.1. Interpersonal Foundations of Stress Appraisals and Goal Engagement

To understand how relational support is linked to long-term well-being, it is first useful to examine its association with how individuals psychologically navigate daily life, shaping cognitive and motivational frameworks during both stressful and aspirational times. Our first set of hypotheses evaluated these mechanisms, examining how SOS and RC support may alter appraisals and self-efficacy beliefs regarding both life stressors and opportunities for growth. The findings suggest that supportive interactions may serve as a vital form of interpersonal scaffolding that shapes how SGD young adults interpret both their vulnerabilities and capacities.

*SOS Support and the Mitigation of Stress.* Our findings revealed that when SGD young adults reported receiving responsive SOS support from close others, they experienced lower subjective impact from stressors, including both general life challenges and identity-specific experiences of stigma, marginalization, or concealment. They also reported greater perceived self-efficacy for coping with and overcoming these stressors. These findings suggest that SOS support—a “safe haven” form of support that promotes thriving through adversity—may reduce cognitive appraisals of threat by expanding perceived coping resources and providing a reliable relational anchor that reduces the perceived severity of stressors. As a result, these stressors may feel less threatening and more manageable.

RC support also uniquely and positively predicted coping self-efficacy. This finding aligns with the theoretical premise that being encouraged and supported in the pursuit of growth cultivates a broader sense of personal mastery, helping individuals feel more capable of handling the challenges that accompany adversity. Because growth and adversity are often intertwined, the benefits of RC support likely extend beyond goal pursuit to coping with stressors as well. Although RC support is conceptualized as facilitating engagement with life opportunities rather than buffering against adversity per se, the pursuit of goals is rarely free of difficulty. Striving toward growth often entails setbacks and obstacles along the way, such that challenge is woven into the process of goal pursuit. Close others who provide effective RC support may therefore help individuals persist through these difficulties and accumulate experiences of navigating hardship successfully. Over time, the mastery cultivated through supported goal pursuit should generalize beyond the domain of growth and contribute to more effective coping with stressors.

*RC Support and the Amplification of Goal Pursuits.* A parallel, distinct pathway emerged in the context of opportunities and growth. When participants reported receiving more responsive RC support, they demonstrated greater focus on pursuing both global life opportunities and identity-specific milestones, such as building confidence and living authentically in their LGBTQ+ identity. RC support also uniquely predicted self-efficacy for goal-attainment, reflecting elevated confidence in their capacity to capitalize on life opportunities for growth. These findings suggest that RC support—involving a “secure base” that promotes thriving through embracing life-opportunities for growth—may accelerate motivation and broaden behavioral horizons. For SGD young adults, identity development often requires high-stakes exploration—such as coming out, dressing authentically, or seeking community—which carries distinct vulnerability. When loved ones act as relational catalysts—showing enthusiasm, helping develop action plans, and celebrating future-oriented identities—they validate these goals and encourage self-discovery (Baron & Collins, 2025). This, in turn, may foster the belief that personal and identity-related aspirations are within reach.

### 4.2. Dual Pathways to Thriving

A central challenge in social support research is explaining how highly correlated support constructs operate uniquely without being redundant. Our attachment-informed theoretical framework—and our theory of thriving through relationships—anticipates substantial shared variance between SOS and RC support, reflecting a common foundation of felt security that underlies both sources of support. This shared variance is theoretically meaningful and expected: individuals who experience high-quality support in one relational context are likely to do so in another, and this common relational foundation is itself an important contributor to thriving. This assumption was well-supported in the current data, which showed that SGD young adults whose close others were available and responsive during times of stress also tended to be supported in their strivings for growth and discovery. In addition, both forms of support were positively correlated with all components of well-being (Table 2), and this overlap accounted for a substantial portion of the variance explained in each outcome. At the same time, our regression, commonality, and latent path analyses moved beyond this shared foundation to demonstrate that each support type also made meaningful and distinct incremental contributions to specific aspects of well-being in theoretically expected ways. These unique contributions provide valuable insight into the possible dual relational pathways to thriving for SGD young adults.

*Source of Strength Support as a Protective Relational Resource.* Our hypotheses regarding SOS support were supported. After controlling for shared contributions with RC support, SOS support uniquely predicted latent Self-integrity, Security, and Growth, but not Identity. These findings are consistent with our conceptualization of SOS as a protective resource linked to resilience-related outcomes. Having a reliable safe haven during adversity, and facilitation of thriving through adversity, may be especially important for fostering the autonomy, clarity, and confidence needed to navigate stressors and stigma (Self-integrity). Similarly, SOS support strengthens personal and social safety by promoting environmental mastery, self-acceptance, and positive relations with others (Security). In challenging social environments, this relational buffer may reinforce a sense of personal worth, providing a foundation for resilience and core aspects of psychological well-being. SOS support also emerged as a significant, but weaker, predictor of latent Growth. This finding is consistent with both attachment theory and our theory of thriving through relationships (Feeney & Collins, 2015a), which suggests that feeling supported during times of stress may do more than just restore safety and stability. SOS support additionally helps people to grow through their adversity and exceed baseline levels of well-being. When close others provide a safe haven during difficult times, they may also help individuals build the confidence needed to take risks, explore new possibilities, and pursue personal growth. In this way, SOS support may indirectly contribute to growth by helping young adults feel grounded enough to move toward new opportunities.

*Relational Catalyst Support and Growth-Oriented Outcomes.* Our hypotheses regarding RC support were largely supported. After controlling for SOS support, RC support uniquely predicted latent Identity, Growth, and Security, but not Self-Integrity. These findings are consistent with our view of RC support as a resource that promotes growth by encouraging goal-directed behavior, validating personal aspirations, and fostering pride in one’s identity.

These findings are also consistent with Baron and Collins’s (2025) perspective on thriving for SGD populations. When close others actively encourage an individual’s goals—including identity-related goals—this support may help cultivate feelings of validation, acceptance, and positive self-regard. For SGD young adults, this active encouragement may be one process through which RC support is associated with greater LGBTQ+ pride and stronger community connection (Identity). Furthermore, by affirming an individual’s potential, relational catalysts may support young adults in clarifying their life direction, aligning goal pursuit with self-improvement and a clearer sense of purpose (Growth). Finally, because relational validation and encouragement are closely tied to experiences of mastering new challenges, cultivating self-acceptance, and building trusting relationships, these forms of support may also co-occur with a broader sense of personal and social safety (Security).

Although we expected RC support to also predict Self-Integrity, this association was not unique after accounting for shared variance with SOS support. At the bivariate level, RC support was positively correlated with components of self-integrity (Table 2), but only SOS accounted for unique variance in the full model. This pattern suggests that while encouragement toward future goals relates to a clearer sense of self, the development of autonomy and self-concept clarity may depend more on the stabilizing presence of a safe haven and facilitator of thriving during times of stress. In this way, SOS support may serve as the primary relational anchor for a coherent and autonomous self-concept. Future longitudinal research is needed to clarify how these two forms of support may work together over time to shape this internal development.

### 4.3. Limitations and Future Directions

Despite the strengths of our study, including a robust sample size and a latent variable framework, several limitations must be noted. First, the data are cross-sectional, which precludes any conclusions regarding causality or temporal ordering. Although our regression and path analyses specify support as a statistical predictor of well-being, these associations are likely bidirectional and shaped, at least in part, by selection processes. For instance, SGD young adults who already possess higher levels of self-acceptance, positive identity, or trusting relationships may be more skilled at soliciting, recognizing, or maintaining relationship partners who act as relational catalysts or sources of strength. Conversely, individuals experiencing lower well-being may have fewer relational resources available to them or may perceive available support less positively. Thus, the observed associations likely reflect dynamic and reciprocal processes between close relationships and well-being rather than one-directional effects. Future research should use longitudinal and daily diary designs to establish temporal precedence, examine within-person change, and disentangle these transactional processes over time. We also note that our SEM was intended to serve as a theory-guided exploratory summary—offering a high-level conceptual synthesis of thriving processes within the SGD community—rather than a definitive, rigidly constrained model. Consequently, both the structural pathways and the measurement model are meant to provide an empirical foundation that inspires future confirmatory work, rather than represent a final, exhaustive account of these relational dynamics and latent components of thriving.

Second, while our sample included diverse sexual and gender identities, just over half of the participants identified as bisexual+. Analyzing SGD participants as a single group was guided primarily by our theoretical framework, which emphasizes the shared experiences of marginalization, resilience, and social safety across the collective SGD community. Because we did not have an a priori theoretical basis to predict divergent pathways between specific identity subgroups, our investigation was designed to capture these overarching relational processes. Methodologically, this approach also aligned with our sample size, which lacked the statistical power necessary to conduct robust subgroup comparisons without risking unstable estimates. Consequently, we could not evaluate distinct pathways for lesbian, gay, or transgender experiences, highlighting the need for larger, targeted samples in future research. Expanded samples would enable researchers to examine how the balance between SOS and RC support might differ across specific identities. In particular, more work is needed to explore these relational processes among transgender and gender-diverse individuals, who frequently navigate unique structural and interpersonal stressors—such as systemic transphobia, legal threats, and navigating medical or social transition. Understanding how different types of relational support buffer these specific challenges or facilitate gender-affirming growth remains a critical next step for the field.

While our sample focused broadly on SGD young adults, future work should also adopt an intersectional approach. The experiences of social support, identity pride, and environmental mastery likely differ substantially based on the intersection of sexual/gender diversity with race, ethnicity, socioeconomic status, and geographic location.

Finally, our work focuses exclusively on receiving social support, leaving the active role of the support-seeker unexplored (Forest et al., 2021). Seeking support can be uniquely challenging for SGD young adults, who must frequently evaluate whether a relationship is safe and affirming before reaching out. This additional cognitive load may inhibit support-seeking and reduce the likelihood of receiving necessary aid. Consequently, interventions targeting support deficits should equally balance teaching effective support-seeking strategies with cultivating safe environments that naturally facilitate these positive exchanges.

## 5. Conclusions

Young adulthood is a critical period for identity development, particularly for SGD young adults who must frequently navigate minority stress. By evaluating the independent contributions of SOS and RC support, this study provides evidence consistent with dual-process models of relational thriving (Baron & Collins, 2025; Feeney & Collins, 2015a). While SOS support acts as a reliable safe haven that fosters resilience and security during adversity, RC support serves as a safe launchpad that promotes identity pride, community connection, and personal growth. Ultimately, ensuring that SGD youth can fully thrive requires close relationships that offer both support functions. Interventions should therefore continue to mitigate the impact of distress while equally focusing on facilitating positive personal growth and empowering SGD young adults to engage in effective support-seeking from affirming networks. Moving forward, these findings advance our theoretical understanding of how distinct relational resources uniquely shape well-being, social safety, and identity development in underrepresented populations.

## Figures and Tables

**Table 1:** Participant Demographic Characteristics.

Variable	*n*	%
**Sexual Orientation**		
Lesbian	30	7.50
Gay	31	7.75
Bisexual+	205	51.25
Queer	23	5.75
Asexual	20	5.00
Multiple labels	63	15.75
Straight	3	0.75
Questioning	4	1.00
Prefers to self-describe	21	5.25
**Gender Identity**		
Man	117	29.25
Woman	217	54.25
Non-Binary	57	14.25
Prefers to self-describe	9	2.25
**Transgender Status**		
Self-identifies as transgender	75	18.75
Does not self-identify as transgender	320	80.00
Prefers not to answer	5	1.25
**Race/Ethnicity**		
Asian	54	13.5
Black	48	12.0
Hispanic/Latino	33	8.25
Middle Eastern or North African	2	0.50
Multiracial	60	15.0
Native Hawaiian or Pacific Islander	1	0.25
Non-Latinx White	201	50.25
Another Race	1	0.25
**Relationship Status**		
One partner	194	48.5
Multiple partners	16	4.0
No partner	190	47.5

*Note. N* = 400.

**Table 2:** Means, standard deviations, and bivariate correlations for all study variables.

Variable	*M*	*SD*	1	2	3	4	5	6	7	8	9	10	11	12	13	14	15	16
1. SOS Support	3.61	0.80																
2. RC Support	3.74	0.77	.89 **															
3. Stressors Global	2.00	0.63	−.32 **	−.27 **														
4. Stressors Identity	1.99	0.83	−.19 **	−.15 **	.83 **													
5. Coping Efficacy	4.07	1.05	.48 **	.49 **	−.32 **	−.15 **												
6. Goals Global	2.66	0.70	.25 **	.32 **	.38 **	.43 **	.25 **											
7. Goals Identity	2.34	1.14	.18 **	.21 **	.40 **	.52 **	.13 **	.79 **										
8. Goals Efficacy	4.18	0.96	.49 **	.53 **	−.25 **	−.06	.80 **	.30 **	.16 **									
9. Autonomy	3.91	0.81	.22 **	.25 **	−.13 *	−.06	.42 **	.09	.01	.42 **								
10. Env. Mastery	3.40	0.98	.42 **	.44 **	−.34 **	−.18 **	.71 **	.24 **	.13 **	.68 **	.39 **							
11. Personal Growth	4.52	0.84	.41 **	.45 **	−.10	.02	.55 **	.30 **	.18 **	.58 **	.42 **	.44 **						
12. Positive Relations	3.84	0.97	.68 **	.66 **	−.33 **	−.15 **	.50 **	.27 **	.18 **	.54 **	.30 **	.60 **	.47 **					
13. Purpose	3.92	0.91	.44 **	.45 **	−.19 **	−.04	.64 **	.25 **	.17 **	.68 **	.38 **	.65 **	.68 **	.53 **				
14. Self-Acceptance	3.45	1.11	.49 **	.50 **	−.22 **	−.08	.69 **	.29 **	.19 **	.70 **	.43 **	.78 **	.54 **	.63 **	.68 **			
15. Self Clarity	3.03	0.92	.40 **	.36 **	−.29 **	−.16 **	.44 **	.02	−.02	.45 **	.51 **	.55 **	.34 **	.50 **	.48 **	.56 **		
16. LGBTQ Pride	3.30	1.16	.41 **	.42 **	.01	.08	.25 **	.43 **	.48 **	.31 **	.22 **	.30 **	.34 **	.43 **	.28 **	.36 **	.27 **	
17. LGBTQ Connect	2.94	1.01	.37 **	.40 **	.02	.10	.22 **	.42 **	.47 **	.27 **	.17 **	.34 **	.28 **	.46 **	.29 **	.35 **	.30 **	.71 **

*Note. M* and *SD* represent the mean and standard deviation, respectively. Coping Efficacy = Self-efficacy for Coping with Stressors. Goals Efficacy = Self-efficacy for Achieving Goals. Self Clarity = Self-Concept Clarity. * *p* < .05. ** *p* < .01.

**Table 3:** Multiple Regression Models (and Commonality Analyses) Predicting Responses to Life Stressors and Opportunities for Growth from Source of Strength (SOS) and Relational Catalyst (RC) Support.

						Commonality Analysis			
Outcome Variable	Predictor	*β*	95% CI	*p*	*R* ^2^	*R*^2^ Common	*R*^2^ Unique	Proportion *R*^2^ Common	Proportion *R*^2^ Unique
Stressors—Global	SOS Support	−.291 **	[−.535, −.207]	.001	.101	.07	.03	.72	.27
	RC Support	.050	[−.110, .231]	.567			.00		.01
Stressors—Identity	SOS Support	−.290 **	[−.506, −.072]	.009	.041	.02	.02	.54	.41
	RC Support	.107	[−.123, .328]	.352			.00		.05
Coping Efficacy	SOS Support	.257 **	[−.051, .446]	.043	.249	.22	.01	.89	.03
	RC Support	.425 **	[.056, .573]	.001			.02		.08
Goals—Global	SOS Support	−.170 *+*	[−.377, −.014]	.066	.109	.05	.01	.48	.07
	RC Support	.446 **	[.304, .681]	<.001			.05		.45
Goals—Identity	SOS Support	−.062	[−.321, .223]	.656	.034	.02	.00	.68	.01
	RC Support	.295 *	[−.056, .509]	.041			.01		.30
Goals Efficacy	SOS Support	.103	[−.138, .310]	.367	.279	.24	.00	.85	.01
	RC Support	.558 ***	[.217, .682]	<.001			.04		.14

*Note. β* = standardized regression coefficient; + *p* < .10. * *p* < .05. ** *p* < .01. *** *p* < .001; CI = confidence interval. Coping Efficacy = Self-efficacy for Coping with Stressors. Goals Efficacy = Self-efficacy for Achieving Goals.

**Table 4:** Multiple Regression Models (and Commonality Analyses) Predicting Thriving Outcomes from Source of Strength (SOS) and Relational Catalyst (RC) Support.

						Commonality Analysis			
Outcome Variable	Predictor	*β*	95% CI	*p*	*R* ^2^	*R*^2^ Common	*R*^2^ Unique	Proportion *R*^2^ Common	Proportion *R*^2^ Unique
Personal Growth	SOS Support	.030	[−.177, .235]	.772	.201	.16	.00	.82	.00
	RC Support	.457 ***	[.208, .636]	<.001			.04		.18
Purpose	SOS Support	.212 +	[−.033, .410]	.060	.206	.18	.01	.89	.03
	RC Support	.326 **	[.048, .509]	.006			.02		.08
Autonomy	SOS Support	−.008	[−.224, .208]	.943	.062	.05	.00	.79	.00
	RC Support	.270 *	[.032, .481]	.019			.01		.21
Environmental Mastery	SOS Support	.190	[−.084, .396]	.122	.198	.17	.00	.88	.02
	RC Support	.378 **	[.050, .549]	.003			.02		.09
Self-Acceptance	SOS Support	.252 +	[−.080, .444]	.060	.261	.23	.01	.89	.03
	RC Support	.490 ***	[.069, .614]	<.001			.02		.09
Positive Relations	SOS Support	.574 ***	[.282, .668]	<.001	.475	.42	.04	.88	.09
	RC Support	.291 **	[.031, .432]	.005			.01		.02
Self-Concept Clarity	SOS Support	.438 ***	[.149, .614]	<.001	.157	.13	.03	.81	.19
	RC Support	.019	[−.226, .258]	.875			.00		.00
LGBTQ Pride	SOS Support	.195	[−.153, .423]	.184	.182	.16	.00	.88	.02
	RC Support	.453 **	[.002, .601]	.003			.02		.10
LGBTQ Connection	SOS Support	.107	[−.169, .339]	.407	.159	.14	.00	.86	.01
	RC Support	.418 **	[.056, .585]	.002			.02		.13

*Note. β* = standardized regression coefficient; + *p* < .10. * *p* < .05. ** *p* < .01. *** *p* < .001; CI = confidence interval.

**Table 5:** Parameter Estimates for the Effects of Source of Strength and Relational Catalyst Support on Latent Components of Well-Being.

Outcome Variable	Indicator/Predictor	*b*	*β*	*z*	*p*
*Factor Loadings*					
Growth	Personal Growth	1.000	.772	—	—
	Purpose	1.178	.844 ***	12.824	<.001
Self-Integrity	Autonomy	1.000	.582	—	—
	Self-Clarity	1.754	.904 ***	7.232	<.001
Security	Positive Relations	1.000	.846	—	—
	Self-Acceptance	1.026	.760 ***	14.884	<.001
	Environmental Mastery	0.828	.696 ***	14.211	<.001
Pride in Identity	LGBTQ Pride	1.000	.844	—	—
	LGBTQ Connection	0.871	.845 ***	13.133	<.001
*Regression Paths*					
Growth	SOS Support	0.193	.241 *	2.019	.044
	RC Support	0.296	.356 **	2.753	.006
Self-Integrity	SOS Support	0.228	.385 ***	3.333	<.001
	RC Support	0.033	.053	0.444	.657
Security	SOS Support	0.426	.416 ***	3.705	<.001
	RC Support	0.381	.358 ***	3.442	<.001
Pride in Identity	SOS Support	0.159	.130	0.971	.332
	RC Support	0.467	.369 **	2.892	.004
*Covariances*					
SOS Support ↔ RC Support		0.556	.895 ***	13.669	<.001
Growth ↔ Self-Integrity		0.097	.431 ***	4.278	<.001
Growth ↔ Security		0.180	.636 ***	6.611	<.001
Growth ↔ Pride in Identity		0.100	.223 **	3.194	.001
Self-Integrity ↔ Security		0.154	.666 ***	5.840	<.001
Self-Integrity ↔ Pride in Identity		0.079	.215 **	3.159	.002
Security ↔ Pride in Identity		0.189	.410 ***	5.518	<.001
Self-Acceptance ↔ Environmental Mastery		0.274	.542 ***	6.464	<.001
Personal Growth ↔ Autonomy		0.110	.313 ***	4.902	<.001
Purpose ↔ Environmental Mastery		0.169	.501 ***	5.204	<.001
Purpose ↔ Self-Acceptance		0.157	.453 ***	4.658	<.001

*Note. N* = 400. *β* = standardized path coefficient. * *p* < .05. ** *p* < .01. *** *p* < .001. SOS Support = Source of Strength Support. RC Support = Relational Catalyst Support. Dashes indicate fixed parameters for which significance tests were not computed.

## Data Availability

The data, analysis code, and survey measures presented in this study are publicly available via the Open Science Framework at https://osf.io/7pcdb/ (accessed on 21 June 2026).

## Notes

1 Gallup (2026) defines Generation Z adults as individuals born between 1997 and 2012 who were age 18 or older at the time of Gallup’s 2025 survey, corresponding to ages 18 to 29 in that report.

2 In this paper, SGD serves as a broad umbrella term that includes people identifying as LGBTQ+ and those with identities that are more fluid, culturally specific, or not represented by acronym-based terminology. This language reflects current trends in both academic research and community usage (Diamond & Alley, 2022; Hammack & Wignall, 2023; National Academies of Sciences, Engineering, and Medicine, 2020) and follows APA protocols for respectful, non-stigmatizing terminology (American Psychological Association, 2023). While inclusive, it is important to note that the term SGD may not align with research questions that reference a specific group or identity (e.g., bisexual or transgender individuals).

3 To address potential exclusion bias, we conducted a sensitivity analysis comparing the 45 excluded participants with the 400 retained participants on key demographic variables. It is worth noting that many excluded participants had incomplete data, which itself may reflect the nature of their exclusion (e.g., failure to complete the survey). Nonetheless, several demographic differences emerged. Compared to retained participants, excluded participants were significantly older (*p* < .001; *M* = 24.2 vs. 22.6), more likely to identify as male (80% vs. 30%; *p* < .001), and less likely to identify as non-binary (0% vs. 14%) or transgender (11% vs. 19%; *p* = .005). Excluded participants also differed significantly in sexual orientation (*p* < .001), with a higher proportion identifying as gay (23% vs. 8%) and straight (25% vs. 1%), and a lower proportion identifying as bisexual (32% vs. 51%). No significant differences were found for racial-ethnic identity (*p* = .796) or student status (*p* = .176). Given that excluded participants were slightly older and more likely to identify as male or gay, mild caution should be exercised when generalizing the current findings across the full demographic spectrum of the SGD young adult population.

4 To ensure the stressor and goal lists were highly relevant and ecologically valid for this population, we conducted a pilot study with a sample of *N* = 142 LGBTQ+ participants (age range 18–25) who completed open-ended prompts regarding the primary stressors and personal goals they encounter in their daily lives. Three independent raters coded these qualitative text responses to identify recurring themes. The final 30 items for the stressor list and 14 items for the goal list utilized in the current study represent the most frequently occurring, high-consensus themes extracted from that thematic analysis.
